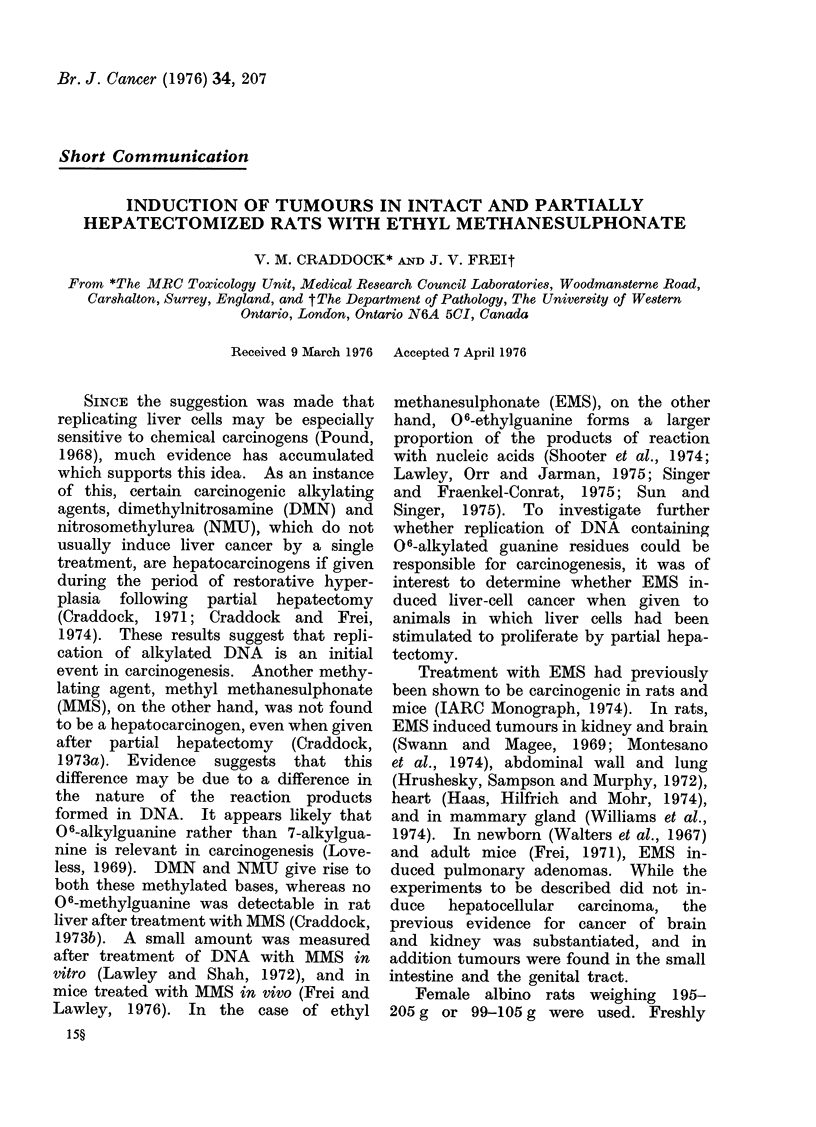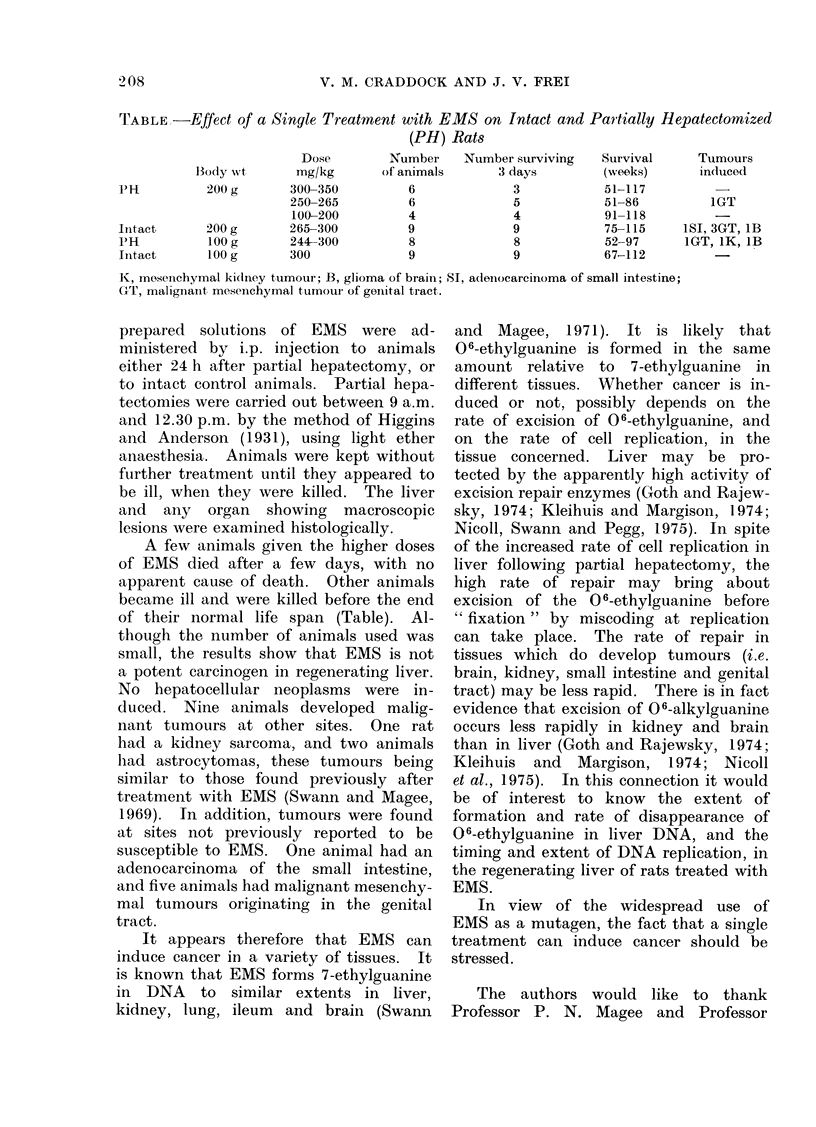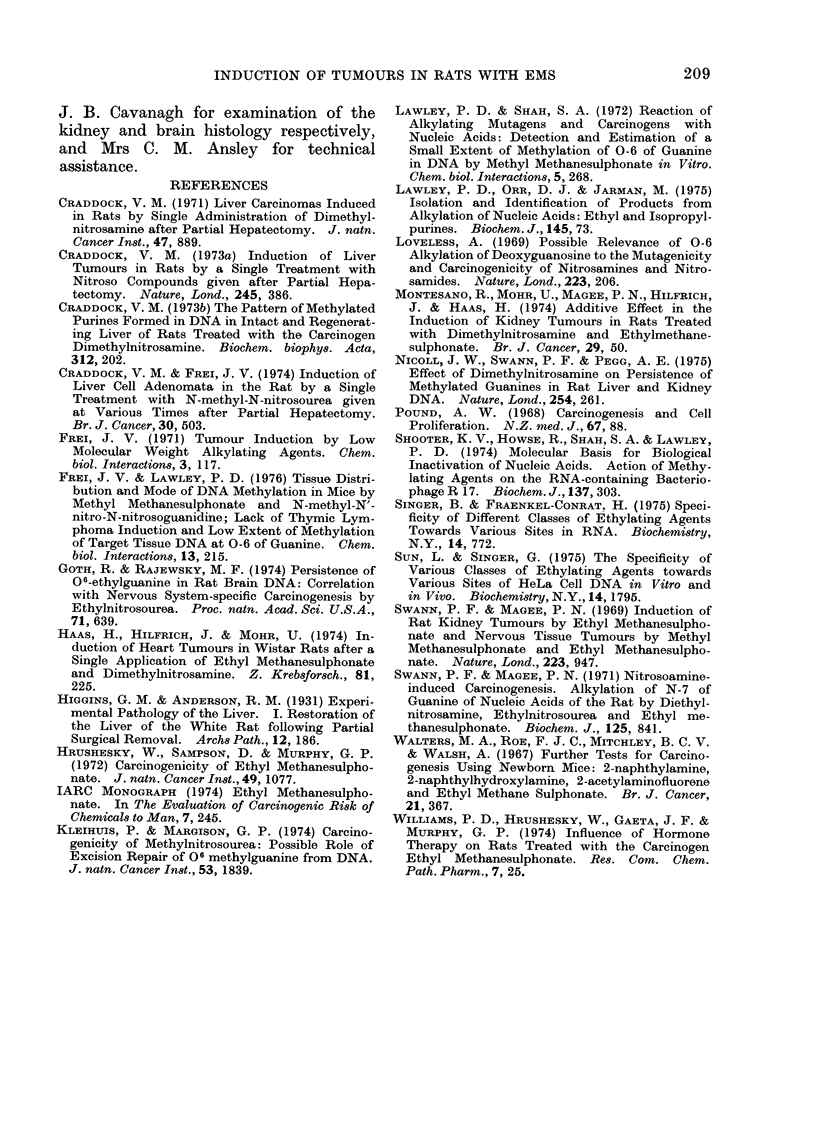# Induction of tumours in intact and partially hepatectomized rats with ethyl methanesulphonate.

**DOI:** 10.1038/bjc.1976.145

**Published:** 1976-08

**Authors:** V. M. Craddock, J. V. Frei


Br. J. Cancer (1976) 34, 207

### *Short Communication*

### INDUCTION OF TUMOURS IN INTACT AND PARTIALLY HEPATECTOMIZED RATS WITH ETHYL METHANESULPHONATE

V. M. CRADDOCK* AND J. V. FREI†

*From *The MRC Toxicology Unit, Medical Research Council Laboratories, Woodmansterne Road, Carshalton, Surrey, England, and †The Department of Pathology, The University of Western Ontario, London, Ontario N6A 5CI, Canada*

Received 9 March 1976 Accepted 7 April 1976

SINCE the suggestion was made that replicating liver cells may be especially sensitive to chemical carcinogens (Pound, 1968), much evidence has accumulated which supports this idea. As an instance of this, certain carcinogenic alkylating agents, dimethylnitrosamine (DMN) and nitrosomethylurea (NMU), which do not usually induce liver cancer by a single treatment, are hepatocarcinogens if given during the period of restorative hyperplasia following partial hepatectomy (Craddock, 1971; Craddock and Frei, 1974). These results suggest that replication of alkylated DNA is an initial event in carcinogenesis. Another methylating agent, methyl methanesulphonate (MMS), on the other hand, was not found to be a hepatocarcinogen, even when given after partial hepatectomy (Craddock, 1973*a*). Evidence suggests that this difference may be due to a difference in the nature of the reaction products formed in DNA. It appears likely that $O^6$-alkylguanine rather than 7-alkylguanine is relevant in carcinogenesis (Loveless, 1969). DMN and NMU give rise to both these methylated bases, whereas no $O^6$-methylguanine was detectable in rat liver after treatment with MMS (Craddock, 1973*b*). A small amount was measured after treatment of DNA with MMS *in vitro* (Lawley and Shah, 1972), and in mice treated with MMS *in vivo* (Frei and Lawley, 1976). In the case of ethyl methanesulphonate (EMS), on the other hand, $O^6$-ethylguanine forms a larger proportion of the products of reaction with nucleic acids (Shooter *et al.*, 1974; Lawley, Orr and Jarman, 1975; Singer and Fraenkel-Conrat, 1975; Sun and Singer, 1975). To investigate further whether replication of DNA containing $O^6$-alkylated guanine residues could be responsible for carcinogenesis, it was of interest to determine whether EMS induced liver-cell cancer when given to animals in which liver cells had been stimulated to proliferate by partial hepatectomy.

Treatment with EMS had previously been shown to be carcinogenic in rats and mice (IARC Monograph, 1974). In rats, EMS induced tumours in kidney and brain (Swann and Magee, 1969; Montesano *et al.*, 1974), abdominal wall and lung (Hrushesky, Sampson and Murphy, 1972), heart (Haas, Hilfrich and Mohr, 1974), and in mammary gland (Williams *et al.*, 1974). In newborn (Walters *et al.*, 1967) and adult mice (Frei, 1971), EMS induced pulmonary adenomas. While the experiments to be described did not induce hepatocellular carcinoma, the previous evidence for cancer of brain and kidney was substantiated, and in addition tumours were found in the small intestine and the genital tract.

Female albino rats weighing 195–205 g or 99–105 g were used. Freshly

TABLE.—*Effect of a Single Treatment with EMS on Intact and Partially Hepatectomized (PH) Rats*

| | Body wt | Dose mg/kg | Number of animals | Number surviving 3 days | Survival (weeks) | Tumours induced |
|---|---|---|---|---|---|---|
| PH | 200 g | 300–350 | 6 | 3 | 51–117 | — |
| | | 250–265 | 6 | 5 | 51–86 | 1GT |
| | | 100–200 | 4 | 4 | 91–118 | — |
| Intact | 200 g | 265–300 | 9 | 9 | 75–115 | 1SI, 3GT, 1B |
| PH | 100 g | 244–300 | 8 | 8 | 52–97 | 1GT, 1K, 1B |
| Intact | 100 g | 300 | 9 | 9 | 67–112 | — |

K, mesenchymal kidney tumour; B, glioma of brain; SI, adenocarcinoma of small intestine; GT, malignant mesenchymal tumour of genital tract.

prepared solutions of EMS were administered by i.p. injection to animals either 24 h after partial hepatectomy, or to intact control animals. Partial hepatectomies were carried out between 9 a.m. and 12.30 p.m. by the method of Higgins and Anderson (1931), using light ether anaesthesia. Animals were kept without further treatment until they appeared to be ill, when they were killed. The liver and any organ showing macroscopic lesions were examined histologically.

A few animals given the higher doses of EMS died after a few days, with no apparent cause of death. Other animals became ill and were killed before the end of their normal life span (Table). Although the number of animals used was small, the results show that EMS is not a potent carcinogen in regenerating liver. No hepatocellular neoplasms were induced. Nine animals developed malignant tumours at other sites. One rat had a kidney sarcoma, and two animals had astrocytomas, these tumours being similar to those found previously after treatment with EMS (Swann and Magee, 1969). In addition, tumours were found at sites not previously reported to be susceptible to EMS. One animal had an adenocarcinoma of the small intestine, and five animals had malignant mesenchymal tumours originating in the genital tract.

It appears therefore that EMS can induce cancer in a variety of tissues. It is known that EMS forms 7-ethylguanine in DNA to similar extents in liver, kidney, lung, ileum and brain (Swann and Magee, 1971). It is likely that $O^6$-ethylguanine is formed in the same amount relative to 7-ethylguanine in different tissues. Whether cancer is induced or not, possibly depends on the rate of excision of $O^6$-ethylguanine, and on the rate of cell replication, in the tissue concerned. Liver may be protected by the apparently high activity of excision repair enzymes (Goth and Rajewsky, 1974; Kleihuis and Margison, 1974; Nicoll, Swann and Pegg, 1975). In spite of the increased rate of cell replication in liver following partial hepatectomy, the high rate of repair may bring about excision of the $O^6$-ethylguanine before " fixation " by miscoding at replication can take place. The rate of repair in tissues which do develop tumours (*i.e.* brain, kidney, small intestine and genital tract) may be less rapid. There is in fact evidence that excision of $O^6$-alkylguanine occurs less rapidly in kidney and brain than in liver (Goth and Rajewsky, 1974; Kleihuis and Margison, 1974; Nicoll *et al.*, 1975). In this connection it would be of interest to know the extent of formation and rate of disappearance of $O^6$-ethylguanine in liver DNA, and the timing and extent of DNA replication, in the regenerating liver of rats treated with EMS.

In view of the widespread use of EMS as a mutagen, the fact that a single treatment can induce cancer should be stressed.

The authors would like to thank Professor P. N. Magee and Professor

J. B. Cavanagh for examination of the kidney and brain histology respectively, and Mrs C. M. Ansley for technical assistance.

REFERENCES

CRADDOCK, V. M. (1971) Liver Carcinomas Induced in Rats by Single Administration of Dimethylnitrosamine after Partial Hepatectomy. *J. natn. Cancer Inst.*, **47**, 889.

CRADDOCK, V. M. (1973*a*) Induction of Liver Tumours in Rats by a Single Treatment with Nitroso Compounds given after Partial Hepatectomy. *Nature, Lond.*, **245**, 386.

CRADDOCK, V. M. (1973*b*) The Pattern of Methylated Purines Formed in DNA in Intact and Regenerating Liver of Rats Treated with the Carcinogen Dimethylnitrosamine. *Biochem. biophys. Acta*, **312**, 202.

CRADDOCK, V. M. & FREI, J. V. (1974) Induction of Liver Cell Adenomata in the Rat by a Single Treatment with N-methyl-N-nitrosourea given at Various Times after Partial Hepatectomy. *Br. J. Cancer*, **30**, 503.

FREI, J. V. (1971) Tumour Induction by Low Molecular Weight Alkylating Agents. *Chem. biol. Interactions*, **3**, 117.

FREI, J. V. & LAWLEY, P. D. (1976) Tissue Distribution and Mode of DNA Methylation in Mice by Methyl Methanesulphonate and N-methyl-N′-nitro-N-nitrosoguanidine; Lack of Thymic Lymphoma Induction and Low Extent of Methylation of Target Tissue DNA at O-6 of Guanine. *Chem. biol. Interactions*, **13**, 215.

GOTH, R. & RAJEWSKY, M. F. (1974) Persistence of $O^6$-ethylguanine in Rat Brain DNA: Correlation with Nervous System-specific Carcinogenesis by Ethylnitrosourea. *Proc. natn. Acad. Sci. U.S.A.*, **71**, 639.

HAAS, H., HILFRICH, J. & MOHR, U. (1974) Induction of Heart Tumours in Wistar Rats after a Single Application of Ethyl Methanesulphonate and Dimethylnitrosamine. *Z. Krebsforsch.*, **81**, 225.

HIGGINS, G. M. & ANDERSON, R. M. (1931) Experimental Pathology of the Liver. I. Restoration of the Liver of the White Rat following Partial Surgical Removal. *Archs Path.*, **12**, 186.

HRUSHESKY, W., SAMPSON, D. & MURPHY, G. P. (1972) Carcinogenicity of Ethyl Methanesulphonate. *J. natn. Cancer Inst.*, **49**, 1077.

IARC MONOGRAPH (1974) Ethyl Methanesulphonate. In *The Evaluation of Carcinogenic Risk of Chemicals to Man*, **7**, 245.

KLEIHUIS, P. & MARGISON, G. P. (1974) Carcinogenicity of Methylnitrosourea: Possible Role of Excision Repair of $O^6$ methylguanine from DNA. *J. natn. Cancer Inst.*, **53**, 1839.

LAWLEY, P. D. & SHAH, S. A. (1972) Reaction of Alkylating Mutagens and Carcinogens with Nucleic Acids: Detection and Estimation of a Small Extent of Methylation of O-6 of Guanine in DNA by Methyl Methanesulphonate *in Vitro*. *Chem. biol. Interactions*, **5**, 268.

LAWLEY, P. D., ORR, D. J. & JARMAN, M. (1975) Isolation and Identification of Products from Alkylation of Nucleic Acids: Ethyl and Isopropylpurines. *Biochem. J.*, **145**, 73.

LOVELESS, A. (1969) Possible Relevance of O-6 Alkylation of Deoxyguanosine to the Mutagenicity and Carcinogenicity of Nitrosamines and Nitrosamides. *Nature, Lond.*, **223**, 206.

MONTESANO, R., MOHR, U., MAGEE, P. N., HILFRICH, J. & HAAS, H. (1974) Additive Effect in the Induction of Kidney Tumours in Rats Treated with Dimethylnitrosamine and Ethylmethanesulphonate. *Br. J. Cancer*, **29**, 50.

NICOLL, J. W., SWANN, P. F. & PEGG, A. E. (1975) Effect of Dimethylnitrosamine on Persistence of Methylated Guanines in Rat Liver and Kidney DNA. *Nature, Lond.*, **254**, 261.

POUND, A. W. (1968) Carcinogenesis and Cell Proliferation. *N.Z. med. J.*, **67**, 88.

SHOOTER, K. V., HOWSE, R., SHAH, S. A. & LAWLEY, P. D. (1974) Molecular Basis for Biological Inactivation of Nucleic Acids. Action of Methylating Agents on the RNA-containing Bacteriophage R 17. *Biochem. J.*, **137**, 303.

SINGER, B. & FRAENKEL-CONRAT, H. (1975) Specificity of Different Classes of Ethylating Agents Towards Various Sites in RNA. *Biochemistry*, N.Y., **14**, 772.

SUN, L. & SINGER, G. (1975) The Specificity of Various Classes of Ethylating Agents towards Various Sites of HeLa Cell DNA *in Vitro* and *in Vivo*. *Biochemistry*, N.Y., **14**, 1795.

SWANN, P. F. & MAGEE, P. N. (1969) Induction of Rat Kidney Tumours by Ethyl Methanesulphonate and Nervous Tissue Tumours by Methyl Methanesulphonate and Ethyl Methanesulphonate. *Nature, Lond.*, **223**, 947.

SWANN, P. F. & MAGEE, P. N. (1971) Nitrosoamine-induced Carcinogenesis. Alkylation of N-7 of Guanine of Nucleic Acids of the Rat by Diethylnitrosamine, Ethylnitrosourea and Ethyl methanesulphonate. *Biochem. J.*, **125**, 841.

WALTERS, M. A., ROE, F. J. C., MITCHLEY, B. C. V. & WALSH, A. (1967) Further Tests for Carcinogenesis Using Newborn Mice: 2-naphthylamine, 2-naphthylhydroxylamine, 2-acetylaminofluorene and Ethyl Methane Sulphonate. *Br. J. Cancer*, **21**, 367.

WILLIAMS, P. D., HRUSHESKY, W., GAETA, J. F. & MURPHY, G. P. (1974) Influence of Hormone Therapy on Rats Treated with the Carcinogen Ethyl Methanesulphonate. *Res. Com. Chem. Path. Pharm.*, **7**, 25.